# Effects of Pomegranate Seed Oil on Insulin Release in Rats with Type 2 Diabetes

**Published:** 2014-03

**Authors:** Ali Akbar Nekooeian, Mohammad Hassan Eftekhari, Soroor Adibi, Abdloreza Rajaeifard

**Affiliations:** 1 Department of Pharmacology, School of Medicine, Shiraz University of Medical Sciences, Shiraz, Iran;; 2 Department of Nutrition, School of Nutrition and Food Sciences, Shiraz University of Medical Sciences, Shiraz, Iran;; 3 Department of Statistics, School of Nutrition and Food Sciences, Shiraz University of Medical Sciences, Shiraz, Iran

**Keywords:** Punicic acid, Diabetes, Insulin

## Abstract

**Background: **Pomegranate seed oil and its main constituent, punicic acid, have been shown to decrease plasma glucose and have antioxidant activity. The objective of the present study was to examine the effects of pomegranate seed oil on rats with type 2 diabetes mellitus.

**Method:** Six groups (n=8 each) of male Sprague-Dawley rats, comprising a control, a diabetic (induced by Streptozocin and Nicotinamide) receiving water as vehicle, a diabetic receiving pomegranate seed oil (200 mg/kg/day), a diabetic receiving pomegranate seed oil (600 mg/kg/day), a diabetic receiving soybean oil (200 mg/kg/day), and a diabetic receiving soybean oil (600 mg/kg/day), were used. After 28 days of receiving vehicle or oils, blood glucose, serum levels of insulin, malondialdehyde, glutathione peroxidase, and lipid profile were determined.

**Results: **The diabetic rats had significantly higher levels of blood glucose, serum triglyceride, low-density lipoprotein cholesterol, total cholesterol, and malondialdehyde and lower levels of serum insulin and glutathione peroxidase. Rats treated with pomegranate seed oil had significantly higher levels of serum insulin and glutathione peroxidase activity, and there were no statistically significant differences in terms of blood glucose between them and the diabetic control group.

**Conclusion: **The findings of the present study suggest that pomegranate seed oil improved insulin secretion without changing fasting blood glucose.

## Introduction

Recently, pomegranate seed oil (PSO) has received considerable dietary attention. The oil’s possible beneficial effects have been attributed to its main bioactive component, punicic acid (*cis9,trans11,cis13*CLnA; conjugated linolenic acid), which constitutes 64-83% of PSO.^1^^,^^2^ Moreover, other CLnA isomers, including α-eleostearic acid and catalpic acid, along with phytosterols, especially β-sitosterol, campesterol, and stigmasterol, are also believed to be involved in the overall health beneficial  effects observed.^2^^,^^3^

Type 2 diabetes is associated with impaired insulin release or insulin resistance, impaired glucose, lipid metabolisms, and increased indices of oxidative stress.^4^^,^^5^ Recent investigations suggest that PSO may reduce the risk of type 2 diabetes by ameliorating high fat diet-induced obesity and insulin resistance.^6^^,^^7^ In line with these findings, pure free isolated punicic acid decreased fasting plasma glucose and improved glucose normalizing ability.^8^ Moreover, PSO was shown to have antioxidant activity^9^^,^^10^ and favorable effects on lipid profiles in hyperlipidemic subjects.^11^

Type 2 diabetes has been induced in rats by the administration of Nicotinamide and Streptozocin.^12^ The model was associated with increased serum glucose, decreased serum insulin, and lipid metabolism disorder.^12^ A literature review demonstrates that there is no published study examining the effects of PSO in an experimental model of diabetes. Therefore, the present study was designed to investigate the effects of PSO on the serum levels of glucose, insulin, malondialdehyde (MDA), glutathione peroxidase, and lipid profile in this model. To account for the difference in energy intake, similar doses of soybean oil (SBO) were also used.

## Material and Methods

*Chemicals*

Streptozotocin (Keocyt, Val de Reuil, France), Nicotinamide (Sigma–Aldrich St Louis, MO, USA), PSO (Techno Parse Company, Tehran, Iran), and unpurified SBO (Animal Food Industry, Shiraz, Iran) were used. The oils were stored in a refrigerator (2-8°C) and protected from light during the study. Streptozocin and Nicotinamide were dissolved in distilled water.

*Ethical Approval*

The study was approved by the Ethics Committee of Shiraz University of Medical Sciences, Shiraz, Iran, and the animals were kept in accordance with the University guidelines on the use and care of animals in research.

*Animals*

Fifty-three healthy male Sprague Dawley rats, weighting 210-270 g, were obtained from the Animal Breeding Center, Shiraz University of Medical Sciences. The animals were housed in polycarbonate cages in standard condition (12 hours light-12 hours dark cycle, temperature of 22-28°C, and humidity of 25-35%) with water and standard rat chow (Behparvar Co., Tehran, Iran) containing the following composition (%): raw protein; 23, raw fat; 3.5-4.5, raw fiber; 4-4.5, ash; 10, calcium; 0.95-1.00, phosphorus; 0.65-0.70, Nacl; 0.50-0.55, lysine; 1.15, methionine; 0.33, methionine+systein; 0.63, threonine; 0.72, tryptophan; 0.25, and humidity; 10. The rat chow and water were available ad libitum.

*Experimental Design and Protocol*

This experimental study was performed at Shiraz University of Medical Sciences in a period from June 2012 to November 2013. Male Sprague-Dawley rats were randomly assigned to control and type 2 diabetic groups. Type 2 diabetes was induced in overnight-fasting animals by single intraperitoneal (IP) injections of Streptozocin (65 mg/kg), 15 min after the IP administration of Nicotinamide (100 mg/kg). Seven days after the injection, rats with a fasting blood glucose level>126 mg/dl were considered type 2 diabetes and those with lower levels were discarded.^12^ The control rats (n=8) were assigned to receive water as vehicle. The type 2 diabetic rats were randomly assigned to the five groups (n=8 each) of a vehicle-treated group receiving 400 mg/kg/day water, a PSO-treated group (200 mg/kg/day), a PSO-treated group (600 mg/kg/day) an SBO-treated group (200 mg/kg/day), and an SBO-treated group (600 mg/kg/day). Water, PSO, and SBO were given by gavage for 28 days. SBO was used to account for energy balance between the two experimental groups. During treatment, the rats were weighed weekly, and the dose of the oils was adjusted accordingly. At the end of the treatment period, the overnight-fasting rats were anaesthetized by a single intraperitoneal injection of Thiopental (70 mg/kg). Blood samples were collected by cardiac puncture and centrifuged at 3000 rpm for 10 min. The sera were collected, divided into micro-tubes, and kept frozen at -70 °C until analysis.

*Biomarker Analysis*

Serum biomarkers, including total cholesterol (TC), high-density lipoprotein cholesterol (HDL-C), low-density lipoprotein cholesterol (LDL-C), triglyceride (TG), glucose, insulin, glutathione peroxidase (GPX), and MDA, were measured. Lipid profiles (TC, HDL-C, LDL-C, and TG) were measured using the photometric method (Pars Azmoon Company, Tehran, Iran). Blood glucose was measured using a glucose analyzer (ACCU-CHEK Active, Roche, Shanghai, China). Serum insulin was assayed using rat ELIZA kits (Mercodia, Sweden). Glutathione peroxides’ activity was measured using the Paglia and Valentines method.^13^ Serum concentration of MDA were measured by the modified thiobarbituric acid method (spectrophotometry); the intra and inter-assay coefficients of variation were 5.5 and 5.9%, respectively.^14^

*Statistical Analysis*

The data, expressed as mean±SD, were first examined for normality of distribution. As they were normally distributed, they were analyzed using the one-way analysis of variance (ANOVA), followed by the Duncan Multiple Range test for pairwise comparisons. A P≤0.05 was considered statistically significant.

## Results

Weight of the animals in the diabetic group receiving vehicle (256.9±6.7 g) was significantly (P=0.0001) lower than that of the control group (294.5±15.2 g). However, there was no significant difference between the weight of the diabetic rats receiving vehicle and those of the diabetic rats receiving 200 mg/kg/day PSO (267.9±17.2 g), 600 mg/kg/day PSO (267.9±17.2 g), 200 mg/kg/day SBO (261.4±6.7 g), or 600 mg/kg SBO (262.9±13.8 g).

**Figure 1:**
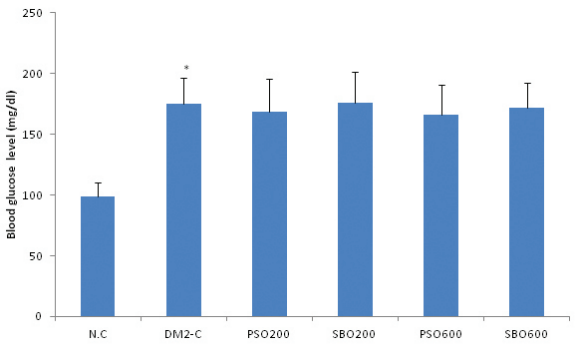
Concentrations (mean±SDM, n=8 each) of fasting blood glucose in the normal control group (N.C), type 2 diabetic control group receiving vehicle (DM2-C), and type 2 diabetic group receiving pomegranate seed oil at 200 mg/kg/day (PSO200) or 600 mg/kg/day (PSO600), or soy bean oil at 200 mg/kg/day (SBO200) or 600 mg/kg/day (SBO600); *Denotes significant (P<0.05) difference from the normal control group

Blood glucose of the diabetic rats receiving vehicle was significantly higher than that of the control rats. However, there was no significant difference between the blood glucose of the animals in the diabetic group receiving PSO (200 or 600 mg/kg) or SBO (200 or 600 mg/kg) (figure 1).

**Figure 2:**
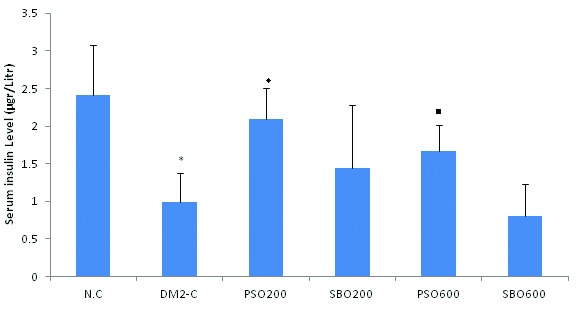
Fasting serum insulin concentrations (mean±SDM, n=8 each) of the normal control group (N.C), type 2 diabetic control group receiving vehicle (DM2-C), and type 2 diabetic group receiving pomegranate seed oil at 200 mg/kg/day (PSO200) or 600 mg/kg/day (PSO600), or soy bean oil  at 200 mg/kg/day (SBO200) or 600 mg/kg/day (SBO600). *Denotes significant (P<0.05) difference from the normal control group; ♦Indicates significant (P<0.05) difference from the DM2-C group; ■Denotes significant (P<0.05) difference from the SBO600-treated group

Serum insulin of the diabetic rats receiving vehicle was significantly lower than that of the controls (P=0.0001) (figure 2). Serum insulin of the diabetic rats treated with PSO (200 mg/kg) was significantly higher than that of the diabetic rats treated with vehicle (P=0.013). Moreover, serum level of insulin in the diabetic rats treated with PSO (600 mg/kg/day) was significantly higher than that of the rats treated with identical doses of SBO (P=0.05) (figure 2).

Serum TG (P=0.001), total cholesterol (P=0.003), and LDL-C (P=0.05) of the diabetic control rats were significantly higher than those of the normal control groups (table 1). However, the serum HDL-C of the diabetic rats treated with vehicle was significantly lower than that of the normal control group (P=0.001). There was no significant difference between the serum levels of TG, total cholesterol of HDL-C, or LDL-C of the groups treated with POS (200 or 600 mg/kg/day) and the diabetic control rats. Moreover, there was no significant difference between the serum levels of TG, total cholesterol, LDL-C, or HDL-C of the diabetic control and the SBO-treated (200 or 600 mg/kg/day) groups (table 1). There was also no significant difference between the effects of the two oils (PSO and SBO) on such variables.

**Table 1:** Serum lipid profile and levels of the indices of oxidative stress (mean±SD, n=8) of the control rats and diabetic rats treated with vehicle, pomegranate seed oil at 200 (PSO1) or 600 mg/kg/day (PSO2), or SBO at 200 mg/kg/day (SBO1) or 600 mg/kg/day (SBO2)

	**TG (mg/dl)**	**TC (mg/dl)**	**HDL-C (mg/dl)**	**LDL-C (mg/dl)**	**MDA (mg/d)**	**GPX (mg/dl)**
	**mean** **±** **SD**	**mean** **±** **SD**	**mean±SD**	**mean±SD**	**mean±SD**	**mean±SD**
Normal control	51.9±4.6	48.3±4.2	34.1±3.7	10.2±1.2	2.1±0.2	470.7±46.6
Diabetic control	67.4±5.4*	56.4±4.6*	28.0±3.2*	12.6±1.7*	2.5±0.2*	368.3±25.9*
Diabetic+PSO1	69.4±8.0	53.6±3.7	28.4±2.2	11. 0±1.5	2.4±0.3	401.8±56.0
Diabetic+SBO1	72.5±6.1	57.4±3.7	27.9±2.2	13.4±1.6	2.5±0.3	373.7±41.8
Diabetic+PSO2	67.3±8.1	58.1±3.6	30.3±2.9	12.9±1.6	2.4±0.2	422.7±39.7♦
Diabetic+SBO2	64.4±8.6	57.6±4.1	28.0±2.1	12.8±1.5	2.5±0.2	341.2±32.6^∆^

TG: Triglyceride; TC: Total cholesterol; HDL-C: High-density lipoprotein cholesterol; LDL-C: Low-density lipoprotein cholesterol; MDA: Malondialdehyde; GPX, glutathione peroxidase; *Denotes significant difference (P<0.05) from the control group; ♦Denotes significant difference (P<0.05) from the diabetic control rats; ^∆^Denotes significant difference (P<0.05) from the diabetic rats receiving 600 mg/kg /day PSO

Serum concentrations of MDA (P=0.04) and GPX (P=0.001) of the diabetic control rats were, respectively, higher and lower than those of the normal control rats (table 1). Neither PSO nor SBO affected serum MDA significantly compared to that of the diabetic control group.  Neither PSO nor SBO (200 mg/kg/day) affected serum GPX significantly compared to that of the diabetic control group. However, the serum levels of the GPX of the group treated with PSO at 600 mg/kg/day was significantly higher than those of the diabetic control group and the diabetic group treated with SBO at 600 mg/kg/day (P=0.005) (table 1).

## Discussion

The findings of the present study indicate that a simultaneous administration of Nicotinamide and Streptozocin resulted in type 2 diabetes mellitus, characterized by increased blood glucose, lipid profile, and oxidative stress as well as with decreased serum insulin. They also showed that PSO increased serum insulin and decreased oxidative stress, without affecting serum lipid profile. 

The present model of diabetes was associated with decreased serum insulin and increased blood glucose. Such findings are similar to those of previous studies using the same model of diabetes^15^ or other models of diabetes.^16^ The model was also associated with increased oxidative stress, which is similar to that of previous studies.^4^^,^^5^

The findings of the present study indicate that PSO did not decrease blood glucose. To the best of our knowledge, this is the first report to evaluate the effect of PSO in an animal model of type 2 diabetes, whereas previous investigations focused mainly on hyperlipidemic^6^^,^^7^^,^^17^or normal animals^8^ or hyperlipidemic subjects.^11^ The lack of PSO effect on blood glucose is similar to that of previous reports.^6^^,^^7^ Such a finding might be due to the presence of insulin resistance, since fat administration is associated with insulin resistance.

The study also showed that PSO increased the serum levels of insulin, but such an increase did not translate into the reduction of blood glucose level. Although the insulin secretion capacity of PSO has not been evaluated previously, improvement in insulin resistance was reported to be significant in hyperlipidemic animals.^6^^,^^7^ The mechanism of a PSO-induced increase in serum insulin is not clear. However, it might not be related to the contribution of fat in that oil, since SBO at similar doses does not change the serum levels of insulin significantly. Moreover, it might be related to the upregulation of PPAR-γ responsive genes as punicic acid, the main ingredient of PSO, was shown to upregulate such genes.^8^

The study showed that PSO did not change lipid peroxidation, as characterized by no change in serum MDA, but reduced the diabetes-induced oxidative stress, characterized by increased serum GPX. This study is the first of its kind to show such an activity for PSO. This finding is in agreement with a previous report demonstrating that punicic acid increased antioxidant activity against sodium arsenite-induced oxidative stress.^9^ Whether or not the effect of PSO to decrease oxidative stress contributes to its PSO-increased insulin activity needs to be examined. Our study also shows that PSO did not change lipid profile in rats with type 2 diabetes. There is, however, no agreement on the effects of PSO on lipid profile, as no changes in TG, PL (phospholipid), HDL-C and TC,^7^^,^^18^ and improvement of TG and TG/HDL-C ratio^11^ have been reported.

The findings of the present study might be interpreted in the light of the fact that there was no previous experience with the use of PSO in experimental models of diabetes, which could limit our ability to choose more appropriate doses of PSO. Therefore, similar studies examining the effects of different doses and treatment protocol of the oil on a wider range of variables would shed more light on the issue.

## Conclusion

The findings of the present study suggest that PSO improved insulin secretion without changing fasting blood glucose

## References

[1] Ahlers NHE, Dennison AC, O'NeillL A (1954). Spectroscopic Examination of Punicic Acid. Nature.

[2] Kaufman M, Wiesman Z (2007). Pomegranate oil analysis with emphasis on MALDI-TOF/MS triacylglycerol fingerprinting. J Agric Food Chem.

[3] Sassano G, Sanderson P, Franx J, Groot P, Straalen JV, Bassaganya-Riera J (2009). Analysis of pomegranate seed oil for the presence of jacaric acid. J Sci Food Agric.

[4] Madkor HR, Mansour SW, Ramadan G (2011). Modulatory effects of garlic, ginger, turmeric and their mixture on hyperglycaemia, dyslipidaemia and oxidative stress in streptozotocin-nicotinamide diabetic rats. Br J Nutr.

[5] Murugan P, Pari L (2006). Antioxidant effect of tetrahydrocurcumin in streptozotocin-nicotinamide induced diabetic rats. Life Sci.

[6] Vroegrijk IO, van Diepen, van den, Westbroek I, Keizer H, Gambelli L (2011). Pomegranate seed oil, a rich source of punicic acid, prevents diet-induced obesity and insulin resistance in mice. Food Chem Toxicol.

[7] McFarlin BK, Strohacker KA, Kueht ML (2009). Pomegranate seed oil consumption during a period of high-fat feeding reduces weight gain and reduces type 2 diabetes risk in CD-1 mice. Br J Nutr.

[8] Hontecillas R, O'Shea M, Einerhand A, Diguardo M, Bassaganya-Riera J (2009). Activation of PPAR gamma and alpha by punicic acid ameliorates glucose tolerance and suppresses obesity-related inflammation. J Am Coll Nutr.

[9] Saha SS, Ghosh M (2009). Comparative study of antioxidant activity of alpha-eleostearic acid and punicic acid against oxidative stress generated by sodium arsenite. Food Chem Toxicol.

[10] Schubert SY, Lansky EP, Neeman I (1999). Antioxidant and eicosanoid enzyme inhibition properties of pomegranate seed oil and fermented juice flavonoids. J Ethnopharmacol.

[11] Mirmiran P, Fazeli MR, Asghari G, Shafiee A, Azizi F (2010). Effect of pomegranate seed oil on hyperlipidaemic subjects: a double-blind placebo-controlled clinical trial. Br J Nutr.

[12] Khalili A, Nekooeian AA, Khosravi MB, Fakher S (2012). Simultaneous renal hypertension and type 2 diabetes exacerbate vascular endothelial dysfunction in rats. Int J Exp Pathol.

[13] Shirwaikar A, Rajendran K, Barik R (2006). Effect of aqueous bark extract of Garuga pinnata Roxb. in streptozotocin-nicotinamide induced type-II diabetes mellitus. J Ethnopharmacol.

[14] Paglia DE, Valentine WN (1967). Studies on the quantitative and qualitative characterization of erythrocyte glutathione peroxidase. J Lab Clin Med.

[15] Mostafavi-Pour Z, Zal F, Monabati A, Vessal M (2008). Protective effects of a combination of quercetin and vitamin E against cyclosporine A-induced oxidative stress and hepatotoxicity in rats. Hepatol Res.

[16] Su HC, Hung LM, Chen JK (2006). Resveratrol, a red wine antioxidant, possesses an insulin-like effect in streptozotocin-induced diabetic rats. Am J Physiol Endocrinol Metab.

[17] Arao K, Wang YM, Inoue N, Hirata J, Cha JY, Nagao K (2004). Dietary effect of pomegranate seed oil rich in 9cis, 11trans, 13cis conjugated linolenic acid on lipid metabolism in obese, hyperlipidemic OLETF rats. Lipids Health Dis.

[18] Yamasaki M, Kitagawa T, Koyanagi N, Chujo H, Maeda H, Kohno-Murase J (2006). Dietary effect of pomegranate seed oil on immune function and lipid metabolism in mice. Nutrition.

